# Self-Control Mediates, and Mobile Phone Dependence Moderates, the Relationship Between Psychological Capital and Attitudes Toward Physical Exercise Among Chinese University Students

**DOI:** 10.3389/fpsyg.2022.888175

**Published:** 2022-05-30

**Authors:** Min Liu, Xinnan Li, Zhonghui He

**Affiliations:** ^1^School of Sports Science, Qufu Normal University, Qufu, China; ^2^School of Physical Education and Art Education, Beijing Technology and Business University, Beijing, China; ^3^Department of Physical Education, Peking University, Beijing, China

**Keywords:** attitudes toward physical exercise, psychological capital, self-control, mobile phone dependence, university students

## Abstract

Physical exercise can improve the psychological capital while the attitude toward physical exercise will affect one’s exercise behavior. However, moderating factors that may influence how physical exercise affects psychological capital remains unknown. We *conducted* a survey of 519 Chinese university students to investigate the mediating role of self-control between attitudes toward physical exercise and psychological capital, and whether this mediating role is moderated by mobile phone dependence. We found that attitudes toward physical exercise had a positive predictive effect on the psychological capital of university students. Besides, self-control mediated the relationship between attitudes toward physical exercise and psychological capital. The influence of self-control on psychological capital was moderated by mobile phone dependence: the influence of self-control on psychological capital decreased with higher mobile phone dependence. Our results suggest that attitudes toward physical exercise can positively predict the psychological capital of university students, with self-control playing a mediating role between them.

## Introduction

The concept of “positive psychology” was first proposed more than 20 years ago (Seligman and Csikszentmihalyi, 2000). At the individual level, positive psychology refers to positive individual characteristics, including love and mission, courage, interpersonal skills, perseverance, tolerance, originality, future consciousness, spirituality, talent, and wisdom. Based on the concept of positive psychology, Luthans and Youssef (2004) developed the concept of “psychological capital” to express the personal motivation tendency generated through a positive psychological structure, which includes four dimensions: self-efficacy, hope, optimism, and resilience. Psychological capital is an important factor for individuals to obtain a competitive advantage, implement positive behavior, and achieve good performance (Zhang et al., 2018).

As a positive psychological trait, psychological capital is based on positive emotions and emotional experience. Physical exercise can improve emotional experiences, leading to a sense of self-efficacy and achievement, as well as psychological capital resources (Luthans and Youssef, 2004; Luthans et al., 2007). Positive psychological resources play an important role in university students’ study, life, and work. Nevertheless, the most recent survey from Conley et al. (2017) showed that university students are at risk for subsequent mental health difficulties and negative psychological states (Conley et al., 2017). The incidence of mental health problems, the severity of symptoms, the use of university psychological counseling services, and duration of treatment have all been increasing (Conley et al., 2017). Thus, university students’ positive psychological resources need to be improved.

Physical exercise positively correlates with university students’ positive psychological characteristics, such as stress management ability, wellbeing, and sense of belonging (Li and Ren, 2009; Pinto et al., 2013). Physical exercise can also improve university students’ intelligence, emotion, interpersonal relationships, and willpower (Guo, 2014). Thus, based on the research paradigm of positive psychology, it is necessary to pay attention to students’ acquisition of positive psychological characteristics and cultivate them through physical exercise. To do that, we first need to clarify the poorly understood relationship between physical exercise and psychological capital, as well as factors affecting that relationship.

Attitudes toward physical exercise refers to the comprehensive expression of the individual attitude toward physical learning and exercise activities. Many studies investigated sports behavior through the theoretical framework of attitude behavior (Bentler and Speckart, 1981; Valois et al., 1988; Daltroy and Godin, 1989). Attitude toward physical exercise will affect physical exercise behavior (Godin and Shephard, 1990): a positive attitude leads to greater exercise behavior among students (Lian, 2000; Li, 2016) and can sustainably drive sports participation (Si, 2005). However, interest in physical exercise among Chinese university students is poor, which mainly manifests as low persistence and lack of exercise habits (Wang, 2016; Huang, 2018).

Physical exercise correlates positively with self-control (Liu, 2016). Motivation and trait self-control regarding physical exercise are predictors of subjective wellbeing (Walid, 2016). Therefore, people who have the ability to control themselves will obtain more psychological capital and derive greater benefit from physical exercise.

At the same time, students’ psychological capital plays an important role in physical exercise and personality development (Yang et al., 2013; Lu and Wang, 2019; Qin and Yuan, 2019). The literature on the relationship between physical exercise and psychological capital has focused on wellbeing and the ability of psychological capital as a regulating role between them (Zhao, 2019; Tan et al., 2020), but it has neglected moderating factors that may influence how physical exercise affects psychological capital.

One such moderating factor may be self-control, which refers to the capacity for altering one’s own responses, especially to bring them into line with standards such as ideals, values, morals, and social expectations, and to support the pursuit of long-term goals (Baumeister et al., 2007). It has been conceived that self-control would represent one of the most adaptive variables of the human psyche (Carver and Scheier, 1998; Tangney et al., 2004). Individuals with high self-control ability can better regulate emotions, improve poor interpersonal skills and subjective wellbeing, and form healthy behavioral patterns, while individuals with low self-control ability experience various problems in life, learning, and social behavior (Tangney et al., 2004; Vohs and Faber, 2007; Moffitt et al., 2011). Individuals with high self-control exhibit greater altruistic behavior and emotional control than those with low self-control (Wills et al., 1995, 2007; Peluso et al., 1999; Duckworth and Kern, 2011). Studies have shown that self-control can improve academic achievement and social behavior (Abedini et al., 2012).

Physical exercise improves self-control (Liu, 2016) and it can create more positive psychological resources (Xie, 2013). According to the control-process theory of self-regulation (Carver and Scheier, 1998), the effectiveness of self-regulation mainly depends upon one’s capacity to pursue clear goals (Walid, 2016). In the case of physical exercise, a positive attitude toward physical exercise, such as consisting an exercise goal, should be associated with high self-control. The energy model theory of self-control, also called the resource-limited model, emphasizes that all self-control behaviors come from and consume a common limited resource (Baumeister et al., 1996). This resource may be more abundant in individuals with greater psychological capital: greater psychological resources mean that more psychological resources are available and that self-control is stronger (Baumeister et al., 2007; Tan and Guo, 2008; Luthans et al., 2015). Therefore, people with high self-control may derive better mental health benefits from physical exercise than those with low self-control. In this way, self-control may mediate the relationship between attitudes toward physical exercise and psychological capital.

A phenomenon that challenges self-control, especially among younger people, is mobile phone use (Wu et al., 2017). Mobile phone dependence, defined as excessive use and an intermittent craving to use a mobile phone (Ezoe et al., 2009), results in various social, behavioral, and affective problems in daily life (Billieux, 2012), is considered as problematic behavior (Toda et al., 2006). Mobile phone dependence occurs 27.4% of teenage users in Hong Kong (Leung, 2008). Younger generations tend to be more dependent on mobile phone (Hakoama and Hakoyama, 2011), because they are at a stage of active development when they show strong curiosity and relatively weak behavioral control (He et al., 2012; Bhise et al., 2014). Mobile phone dependence among students has been linked to poor academic performance, negative emotions, depression, social anxiety, and insomnia (Lepp et al., 2015; Haruka et al., 2017; Aleksandar et al., 2018; Huang et al., 2019). Self-control can significantly affect risk of mobile phone dependence (Kim et al., 2018; Zhang et al., 2019), with the two factors related negatively to each other (Cho et al., 2017; Han et al., 2017; Liu et al., 2018). Previous findings indicated that self-control and self-regulation were the main predictors of mobile phone problematic use among university students (Pourrazavi et al., 2014).

According to Khang et al. (2013), it is suggested that people with poor self-control are vulnerable to pathological use of mobile phones, which may indicate that there is a link between psychological capital and excessive use of mobile phones. It may be that physical exercise can help restore the psychological capital needed to bolster self-control, which may in turn reduce the risk of mobile phone dependence (Yang et al., 2019). Drawing from resource protection theory (Alarcon et al., 2011), we hypothesized that self-control can act as a positive energy source to stimulate regular physical exercise and thereby buffer the negative effect of mobile phone dependence on psychological capital.

**Figure 1:**
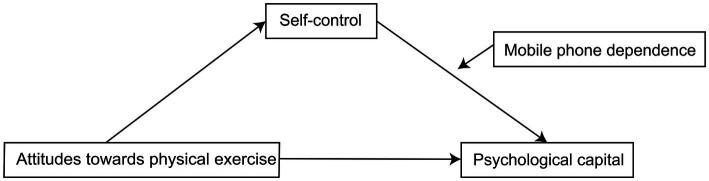
Relationships among the concepts of the study.

To test this hypothesis, we examined here whether self-control mediated the relationship between attitudes toward physical exercise and psychological capital, and whether mobile phone dependence moderated the relationship between self-control and psychological capital (Figure 1).

## Materials and Methods

### Study Subjects

Using the convenience sampling method, we selected 800 first- and second-year students from Beijing Technology and Business University in China to participate in the study. Of them, 800 filled the study questionnaire, 180 questionnaires were discarded because they were not rigorously filled out, for example, multiple questions were marked with two or more answers, and another 101 questionnaires were excluded because more than 10% of items were not answered. Finally, 519 valid questionnaires were obtained, corresponding to an effective response rate of 86.5%. Among the participants, there were 187 men (36.0%) and 332 women (64.0%); 257 subjects (49.5%) were studying liberal arts and 262 (50.5%) sciences. The average age was 19.95 ± 1.34 years.

### Psychological Measurements

#### Physical Exercise Attitude Scale

The physical exercise attitude scale developed by Mao (2003) was applied. The development of this scale conformed to the standard procure of making of psychological scale, which demonstrated accepted internal consistence, content validity, and construct validity by psychometric analysis (Mao, 2003). This scale includes 70 items covering eight dimensions: behavior attitude, goal attitude, behavior cognition, behavior habit, behavior intention, emotional experience, behavior control, and subjective standard. A Likert 7-point scoring method was used, where 1 indicated “total non-compliance” and 7 “full compliance.” Higher score translated to more positive attitude toward exercise. Cronbach’s *α* for the scale in this study was 0.82.

#### Psychological Capital Scale

The positive psychological capital questionnaire developed by Zhang et al. (2010) was applied, which includes 26 items covering four dimensions: self-efficacy, optimism, hope, and resilience. The questionnaire adopted a Likert 7-point scoring method, with 1 point indicating “completely non-conforming” and 7 points “fully conforming.” The higher the score was, the greater the overall psychological capital. Cronbach’s *α* for this scale was 0.90.

#### Self-Control Scale

The self-control scale revised by Tan and Guo (2008) was used to measure individual self-control ability. This scale was revised based on the self-control scale developed by Tangney et al. (2004). The scale has 19 items covering five dimensions: impulse control, healthy habits, resisting temptation, focusing on work, and controlling entertainment. The scoring used a Likert 7-point rating scale, with 1 meaning “completely inconsistent” and 7 “fully consistent.” The higher the total score was, the worse the trait self-control. Cronbach’s *α* for this scale was 0.75.

#### Mobile Phone Addiction Index

This scale was developed by Leung (2008) and translated into Chinese by Huang et al. (2014). It has 17 items belonging to four dimensions: out of control, withdrawal, evasion, and inefficiency. The scoring used a Likert 7-point rating scale, with 1 meaning “almost none” and 7 “always.” Higher scores indicate greater dependence on mobile phones. Cronbach’s *α* for this scale was 0.84.

### Survey Procedure

Physical education professors received rigorous training to perform the test. After obtaining informed consent from each subject, group testing was performed in each school. The questionnaire was anonymous and administered during physical education class time. The questionnaire included 35 questions, and it took students about 20 min to complete.

### Data Analysis

The anonymous method and reverse scoring of some items were used to control the measurement process. The common method deviation after data collection was tested using the Harman single-factor model (Zhou and Long, 2004). Non-rotating principal component analysis was conducted for all items at the same time.

SPSS software version 21.0 and Mplus software version 7.0 were used to analyze the data.

The non-parametric percentile bootstrap method (Berrar, 2019) with deviation correction was used to test the mediating effect of self-control. The results of the variance test showed significant differences in mean psychological capital with different levels of leadership experience, major and family economic status of students. Therefore, we controlled these three variables in subsequent analysis.

According to the methods of Wen and Ye (2014), all predictive variables were standardized, and leadership experience, major, and family economic status were controlled. The parameters of the three regression models were estimated as follows: model 1 tested the regulatory effect of the mobile phone addiction index on the relationship between attitudes toward physical exercise and psychological capital; model 2 tested the moderating effect of the mobile phone addiction index on the relationship between attitudes toward physical exercise and self-control; and model 3 tested the ability of attitudes toward physical exercise, self-control, and mobile phone addiction index to predict psychological capital.

## Results

### Common Method Deviation Test

The results showed 24 factors with eigenvalues greater than 1. The first extracted factor explained 24.2% of the total variation, which was lower than the critical value of 40%. This indicated that common method deviation did not have a significant impact on the results.

### Correlation Matrix of Attitudes Toward Physical Exercise, Self-Control, Mobile Phone Addiction, and Psychological Capital

Table 1 lists the mean, standard deviation, and Pearson’s product–moment correlation matrix of each variable. Attitudes toward physical exercise showed a weak negative correlation with self-control (*r* = −0.243, *p* < 0.01) and mobile phone addiction index (*r* = −0.108, *p* < 0.05), while it positively correlated with psychological capital (*r* = 0.487, *p* < 0.01). Self-control showed a moderate positive correlation with mobile phone addiction index (*r* = 0.399, *p* < 0.01) but negative correlation with psychological capital (*r* = −0.277, *p* < 0.01). Mobile phone addiction index and psychological capital showed a weak negative correlation with each other (*r* = −0.186, *p* < 0.01).

**Table 1:** Mean, standard deviation (SD), and Pearson product–moment correlation matrix of study variables based on 519 questionnaires.

Study variable	Attitudes toward physical exercise	Self-control	Mobile phone addiction index	Psychological capital	Grade	Gender
Attitudes toward physical exercise	–					
Self-control	−0.243^**^	–				
Mobile phone addiction index	−0.108^*^	0.399^**^	–			
Psychological capital	0.487^**^	−0.227^**^	−0.186^**^	–		
Grade	−0.027	0.089^*^	−0.019	0.006	–	
Gender	−0.006	−0.005	0.114^**^	0.068	−0.001	–
Mean	3.11	2.80	2.55	4.70	1.61	1.64
SD	0.496	0.451	0.704	0.608	0.495	0.481

* *p* < 0.05;

** *p* < 0.01.

### The Mediating Effect of Self-Control

The direct effect of attitudes toward physical exercise on psychological capital was 0.52 [95% confidence interval (CI) 0.42–0.61], indicating that attitudes toward physical exercise had a significant positive effect on psychological capital (Table 2). The effect for self-control as an intermediary variable of attitudes toward physical exercise on psychological capital was 0.03 (95% CI 0.01–0.07). Therefore, self-control played a significant intermediary role in the relationship between attitudes toward physical exercise and psychological capital. The proportion of intermediary effect in the total effect was 5.90%. The total effect for attitudes toward physical exercise on psychological capital was 0.55 (95% CI 0.46–0.64).

**Table 2:** Effect sizes and 95% confidence intervals (CI) from non-parametric percentile bootstrapping.

Path	Effect size	Standard error	95% CI	
			Lower limit	Upper limit
Attitudes toward physical exercise → Psychological capital (direct effect)	0.52	0.05	0.42	0.61
Attitudes toward physical exercise →Self-control → Psychological capital (indirect effect)	0.03	0.01	0.01	0.07
Total effect	0.55	0.05	0.46	0.64

### The Moderating Effect of Mobile Phone Dependence on Self-Control

We observed that attitudes toward physical exercise positively predicted psychological capital (*b* = 0.39, *p* < 0.01), but the interaction between attitudes toward physical exercise and mobile phone addiction index did not predict psychological capital (*b* = 0.09, *p* > 0.05; Table 3). Besides, attitudes toward physical exercise negatively predicted self-control (*b* = −0.40, *p* < 0.01) but positively predicted psychological capital (*b* = 0.42, *p* < 0.01). The interaction between self-control and the mobile phone addiction index also predicted psychological capital (*b* = −0.13, *p* < 0.05).

**Table 3:** Predictive ability of the study variables based on non-parametric percentile bootstrapping.

Predictive variable	Model 1: Psychological capital			Model 2: Self-control			Model 3: Psychological capital		
	*b*	SE	*t*	*b*	SE	*t*	*b*	SE	*t*
Attitudes toward physical exercise	0.39^***^	0.16	2.98	−0.40^***^	0.13	−2.85	0.42^***^	0.05	11.06
Mobile phone addiction index	−0.21	0.19	−0.96	0.03	0.15	0.11			
Attitudes toward physical exercise * mobile phone addiction index	0.09	0.06	0.37	0.39	0.05	1.49			
Self-control							−0.02	0.07	−0.36
Self-control * mobile phone addiction index							−0.13^**^	0.01	−2.56
Student cadre experience	−0.12^***^	0.05	−3.30	−0.04	0.04	−0.94	−0.12^***^	0.05	−3.35
Academic major	0.07^*^	0.04	1.83	−0.01	0.04	−0.31	0.07^*^	0.04	1.79
Family economic status	−0.19^***^	0.04	−4.97	0.03	0.03	0.86	−0.18^***^	0.04	−4.91
*R* ^2^	0.32			0.21			0.32		
*F*	39.27			22.12			39.89		

* *p* < 0.05;

** *p* < 0.01;

*** *p* < 0.001.

According to the above results, we found that attitudes toward physical exercise, self-control, mobile phone addiction index, and psychological capital constitute a moderated mediation model, in which the mobile phone addiction index moderates the impact of self-control on psychological capital.

**Figure 2:**
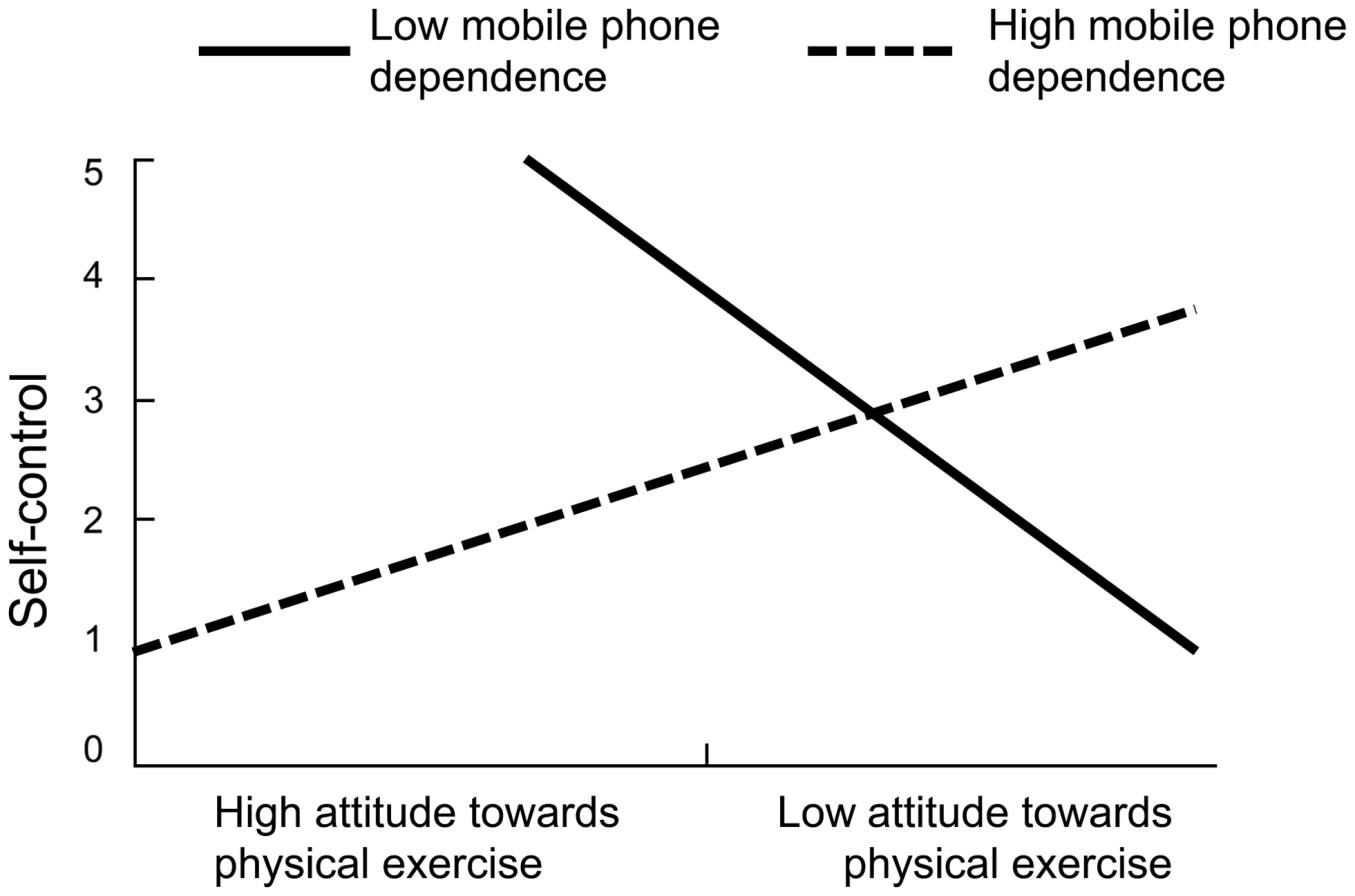
The moderating effect of mobile phone dependence on self-control.

To further analyze the regulatory effect of mobile phone dependence, students were stratified into low or high groups based on whether they were at least one standard deviation below or above the mean value on the mobile phone addiction index, and a simple slope analysis was performed. When the mobile phone addiction index was low (mean −1 SD), self-control showed a significant decreasing trend with increasing physical exercise attitude score (*b* = −1.20, *t* = −1.71, *p* < 0.1; Figure 2). When the mobile phone addiction index was high (mean +1 SD), self-control showed a non-significant upward trend with increasing physical exercise attitude score (*b* = 0.57, *t* = 0.52, *p* > 0.05). In other words, the impact of attitudes toward physical exercise on self-control was higher when mobile phone dependence was low.

## Discussion

We examined the role of self-control on the impact of university students’ attitudes toward physical exercise on psychological capital by conducting a survey among students from Beijing Technology and Business University. Similar to previous studies (Oaten and Cheng, 2006; Gailliot, 2007; Muraven, 2010), we found that attitudes toward physical exercise can positively predict psychological capital (Yang et al., 2013; Zhao, 2014). The characteristics of positive psychological could be an embodiment of positive attitudes toward physical exercise. For example, exercise perseverance is a person’s ability to overcome barriers that inhibit their participation in and physical exercise (Zhu et al., 2001), such as injuries in sports, failure in competition, or lack of cooperation among teammates. Trough physical exercise, students learn the perseverance and a never-give-up attitude and gain the psychological capital.

We further showed that the relationship between attitudes toward physical exercise and psychological capital may be mediated by self-control: we found that self-control can be improved through physical exercise, and previous research found that physical exercise can improve resilience and self-control, such as long-term aerobic exercise (Oaten and Cheng, 2006; Xie, 2013) or group activities like football or basketball (Davis et al., 2016). Our results, together with the literature, suggest that long-term regular physical exercise can help university students enhance their willpower, cultivate optimism, and accumulate more positive “psychological capital.” One way for university students to improve their self-control is to maintain a good level of physical activity.

In our sample, the influence of attitudes toward physical exercise on self-control was higher when the score of mobile phone addiction index was lower. Students who were not addicted to mobile phones had greater self-control when their attitudes toward physical exercise were more positive. In contrast, the attitudes toward physical exercise of students who were addicted to mobile phones did not strongly impact their self-control. This may be because addiction to mobile phones is associated with an increase in sedentary behavior resulting in poor physical activity (Barkley and Lepp, 2016), which means that the time and willingness to participate in physical exercise are reduced, but it had no significant effect on self-control. Participating in sports reduces time and energy invested in mobile phones, which also reduces the possibility of mobile phone addiction. Therefore, schools can consider improving university students’ enthusiasm for, and participation in, physical activities in order to reduce their dependence on mobile phones and mitigate negative emotions (Huo, 2011), such as holding university basketball games.

The results of the present study may help clarify how university students cultivate positive psychological capital. On the one hand, with improvement of the attitude toward physical exercise, the students’ ability of self-control enhances, and their positive psychological capital enhances consequently. On the other hand, due to the increase of self-control, the phenomena of mobile phone dependence decrease, by which positive psychological capital would be strengthened gradually. Our work has practical significance for improving positive psychological effect and self-control, and it provides a theoretical basis for helping students cultivate a healthy lifestyle and engage in physical exercise. To improve positive psychological capital, we can improve students’ capacity for self-control through exercise, positive psychological suggestion, and listening to music. Educators should pay attention to the role of positive psychological education in improving students’ healthy personality and physical development, and one way to do this is to provide more sports programs.

Our study presents some limitations. Self-control appears to mediate only partly the relationship between attitudes toward physical exercise and psychological capital, so other mediating variables need to be considered in the future. At the same time, studies should examine the effects of specific sports interventions on specific groups, such as those strongly dependent on mobile phones or those with low self-control, in order to verify and optimize the positive psychologic benefits for students.

## Conclusion

Our study shows that attitudes toward physical exercise can positively predict university students’ psychological capital, with self-control playing a mediating role between the two factors. Mobile phone addiction can weaken the positive impact of attitudes toward physical exercise on student’s psychological capital, so interventions are needed to mitigate it.

## Data Availability Statement

The raw data supporting the conclusions of this article will be made available by the authors, without undue reservation.

## Author Contributions

ML designed and directed the project. XL helped to supervise the project. ZH verified the analytical methods. All authors contributed to the article and approved the submitted version.

## Funding

This work was supported by 2018 Beijing Social Science Foundation Project (18YTC022), Beijing General Project of Education Commission (SM201910011002), and Construction of Science and Technology Innovation Service Capability-basic Scientific Research Operating Fee-The Relationship between College Students’ Proactive Personality and Academic Performance of Different Sports Groups: The Mediating Effect of Self-Efficacy (pxm2018_014213_000033).

## Conflict of Interest

The authors declare that the research was conducted in the absence of any commercial or financial relationships that could be construed as a potential conflict of interest.

## Publisher’s Note

All claims expressed in this article are solely those of the authors and do not necessarily represent those of their affiliated organizations, or those of the publisher, the editors and the reviewers. Any product that may be evaluated in this article, or claim that may be made by its manufacturer, is not guaranteed or endorsed by the publisher.
